# Crystal structures and supra­molecular features of 9,9-dimethyl-3,7-di­aza­bicyclo­[3.3.1]nonane-2,4,6,8-tetra­one, 3,7-di­aza­spiro­[bi­cyclo­[3.3.1]nonane-9,1′-cyclo­penta­ne]-2,4,6,8-tetra­one and 9-methyl-9-phenyl-3,7-di­aza­bicyclo­[3.3.1]nonane-2,4,6,8-tetra­one di­methyl­formamide monosolvate

**DOI:** 10.1107/S2056989017009458

**Published:** 2017-06-30

**Authors:** Sergey Z. Vatsadze, Marina A. Manaenkova, Evgeny V. Vasilev, Nikolai U. Venskovsky, Victor N. Khrustalev

**Affiliations:** a Department of Chemistry, Moscow State University, 1 Leninskie Gory, Moscow 119991, Russian Federation; b Inorganic Chemistry Department, Faculty of Science, Peoples’ Friendship University of Russia (RUDN University), 6 Miklukho-Maklay St., Moscow 117198, Russian Federation

**Keywords:** crystal structure, alcaloides, di­aza­bicyclo­[3.3.1]nona­nes, tetra­ones, supra­molecular chemistry, hydrogen-bonding inter­actions, crystal structure

## Abstract

Compounds (I), C_9_H_10_N_2_O_4_, (II), C_11_H_12_N_2_O_4_, and (III), C_14_H_12_N_2_O_4_·C_3_H_7_NO represent 9,9-disubstituted-3,7-di­aza­bicyclo­[3.3.1]nonane-2,4,6,8-tetra­one deriv­atives with very similar mol­ecular geometries for the bicyclic framework: the dihedral angle between the planes of the imide groups is 74.87 (6), 73.86 (3) and 74.83 (6)° in (I)–(III), respectively. The dimethyl derivative (I) is positioned on a crystallographic twofold axis and its overall geometry deviates only slightly from idealized *C*_2v_ symmetry. The spiro-cyclo­pentane derivative (II) and the phen­yl/methyl analog (III) retain only inter­nal *C*_s_ symmetry, which in the case of (II) coincides with crystallographic mirror symmetry. The cyclo­pentane moiety in (II) adopts an envelope conformation, with the spiro C atom deviating from the mean plane of the rest of the ring by 0.548 (2) Å. In compound (III), an N—H⋯O hydrogen bond is formed with the di­methyl­formamide solvent mol­ecule. In the crystal, both (I) and (II) form similar zigzag hydrogen-bonded ribbons through double inter­molecular N—H⋯O hydrogen bonds. However, whereas in (I) the ribbons are formed by two *trans*-arranged O=C—N—H amide fragments, the amide fragments are *cis*-positioned in (II). The formation of ribbons in (III) is apparently disrupted by participation of one of its N—H groups in hydrogen bonding with the solvent mol­ecule. As a result, the mol­ecules of (III) form zigzag chains rather than the ribbons through inter­molecular N—H⋯O hydrogen bonds. The crystal of (I) was a pseudo-merohedral twin.

## Abstract 2

The crystal structures of three 9,9-disubstituted-3,7-di­aza­bicyclo­[3.3.1]nonane-2,4,6,8-tetra­ones and their supra­molecular features were studied by X-ray diffraction.

## Chemical context   

Di­aza­bicyclo­nonane-tetra­ones are used in the synthesis of the sparteine subgroup of lupine alcaloids (Norcross *et al.*, 2008 ▸) and are precursors in obtaining 3,7-di­aza­bicyclo­[3.3.1]nona­nes which have been studied in computer models as serine protease inhibitors (Vatsadze *et al.*, 2016 ▸). They also have value as building blocks in the design of other biologically active compounds (Kudryavtsev *et al.*, 2014 ▸), and in the synthesis of imaging agents for positron emission tomography (Medved’ko *et al.*, 2016 ▸). In addition, they are good chelating ligands for 3*d* transition metals (Vatsadze *et al.*, 2005 ▸) including Cu (Vatsadze *et al.*, 2014 ▸).
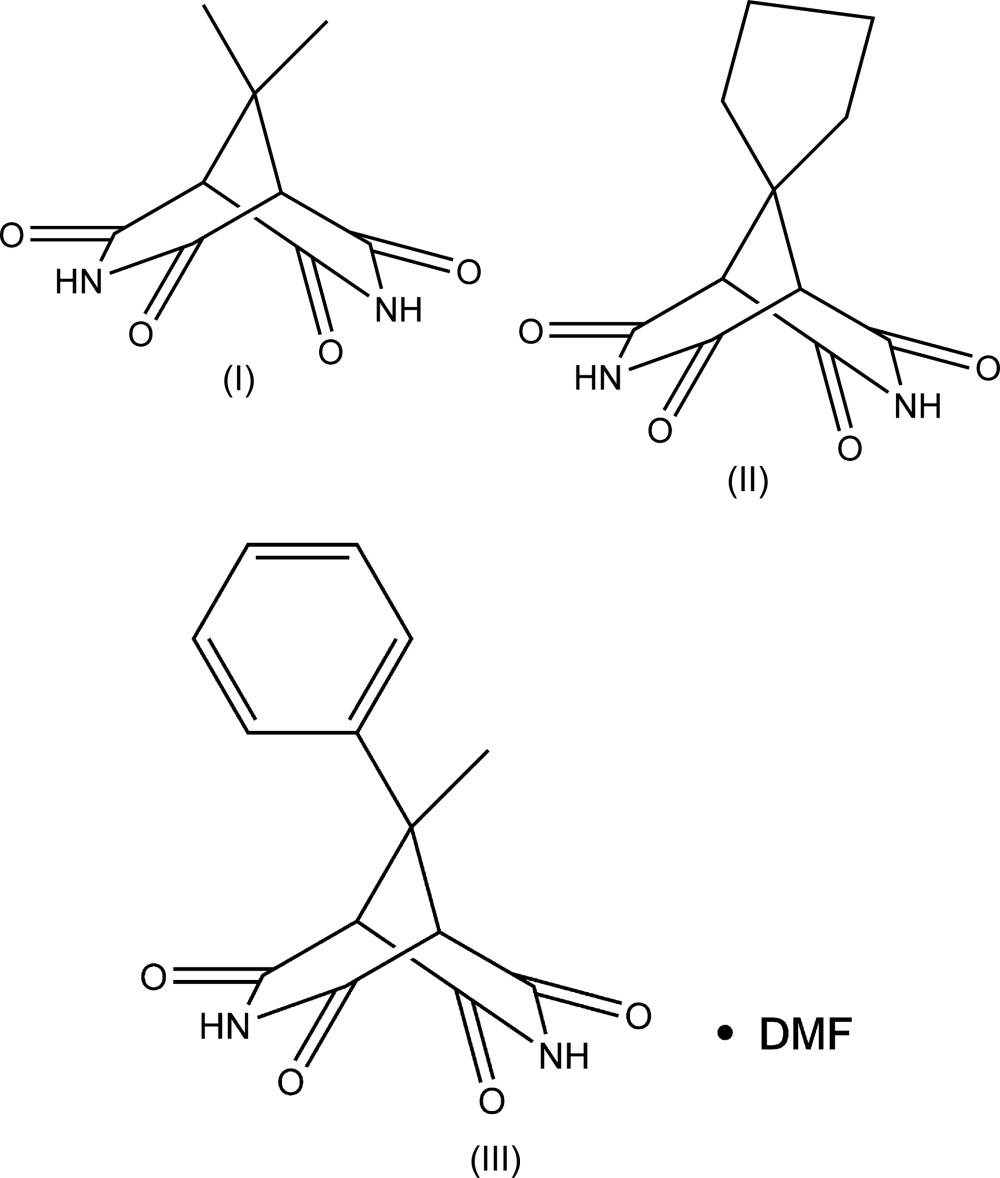


However, the crystal structures of this class of compounds have not been adequately characterized so far, as shown by a small number (eight) of similar structures found in the Cambridge Structural Database (CSD; Groom *et al.*, 2016 ▸). Moreover, their ability to form different supra­molecular structures depending on the substituents at the 9-position in the heterocycle, which we report in this work, has not been not reported before. A search in the CSD for the substructure 3,7-di­aza-2,4,6,8-tetra­oxobi­cyclo­[3.3.1]nonane yielded eight hits. Although there is a similarity in chemical structure of known related compounds (Horlein *et al.*, 1981 ▸; Norcross *et al.*, 2008 ▸), their supra­molecular features are significantly different because of the impact of substituents and solvatation.

In this work, we have synthesized three 9,9-disubstituted-3,7-di­aza­bicyclo­[3.3.1]nonane-2,4,6,8-tetra­ones and show how groups bound to C9 as well as the presence of solvate mol­ecules affect their ability to form different hydrogen-bonding systems.

## Structural commentary   

**Figure 1:**
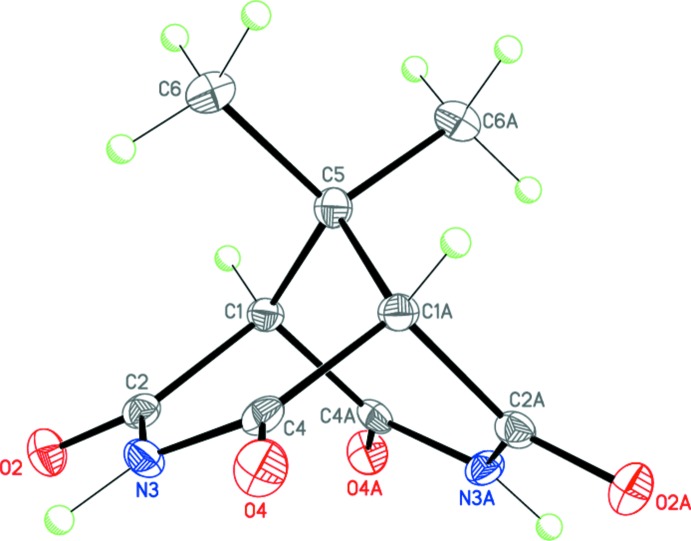
The mol­ecular structure of (I). Displacement ellipsoids are shown at the 50% probability level. H atoms are presented as small spheres of arbitrary radius. [Symmetry code: (A) 1 + *x*, *y*, −*z* + 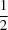.]

Compounds (I), C_9_H_10_N_2_O_4_, (II), C_11_H_12_N_2_O_4_, and (III), C_14_H_12_N_2_O_4_·C_3_H_7_NO represent 9,9-disubstituted-3,7-di­aza­bicyclo­[3.3.1]nonane-2,4,6,8-tetra­one derivatives and have very similar mol­ecular geometries (Figs. 1 ▸–3 ▸ ▸). In general, the 3,7-di­aza­bicyclo­[3.3.1]nonane-2,4,6,8-tetra­one skeleton exhibits idealized *C*_2v_ (*mm*2) symmetry. The mol­ecule of (I), containing two 9-methyl substituents, occupies a special position on a twofold axis [*C*_2_ (2)], and its geometry deviates only slightly from the perfectly symmetrical *C*_2v_. As a result of the presence of spiro-9-cyclo­pentane [in the case of (II)] and 9-phenyl and 9-methyl [in the case of (III)] substituents, the overall symmetry of these mol­ecules decreases to *C*_s_ (*m*). However, in the crystal, the intrinsic *C*_s_ symmetry remains only for the mol­ecule of (II), which occupies a special position on a mirror plane. Compound (III) crystallizes as a dimethyl formamide monosolvate, with the main mol­ecule occupying a general position.

The two imide fragments in the mol­ecules of (I)–(III) are almost planar (r.m.s. deviations are 0.013, 0.009 and 0.009/0.036 Å, respectively). The dihedral angles between the imide planes are 74.87 (6), 73.86 (3) and 74.83 (6)° for (I)–(III), respectively. Moreover, the four carbonyl carbon atoms in (I)–(III) are each coplanar with r.m.s. deviations of 0.018, 0.000, and 0.031 Å, respectively; the bridged carbon atom lies by 1.854 (3), 1.846 (1), and 1.858 (2) Å, respectively, above this plane in (I)–(III). The cyclo­pentane substituent in (II) adopts an envelope conformation, with the C6 spiro-carbon atom deviating from the mean plane through the other ring atoms by 0.548 (2) Å.

**Figure 3:**
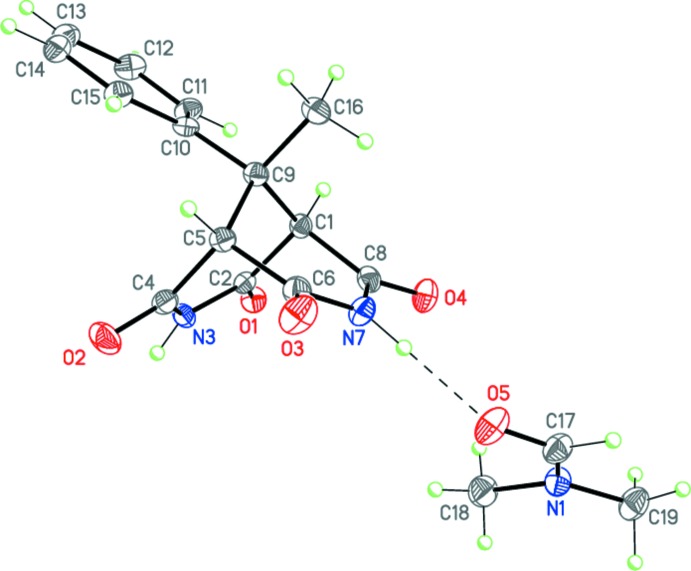
The mol­ecular structure of (III)·DMF. Displacement ellipsoids are shown at the 50% probability level. H atoms are presented as small spheres of arbitrary radius. Dashed line indicates the intra­molecular N—H⋯O hydrogen bond.

Importantly, in (III) the main mol­ecule forms a strong N7—H7⋯O5 hydrogen bond with the dimethyl formamide solvate mol­ecule (Table 3 ▸, Fig. 3 ▸).

## Supra­molecular features   

In general, any compound of type (I)–(III) could form up to six inter­molecular hydrogen bonds utilizing two hydrogen-bond donor NH groups and four hydrogen-bond acceptor carbonyl oxygen atoms. In the literature, even the unsubstituted analogue (refcode GOHHER; Norcross *et al.*, 2008 ▸) shows only four inter­molecular hydrogen bonds involving both imide fragments of bis­pidintetra­one with the formation of an infinite three-dimensional hydrogen-bonded network. If one of the nitro­gen atoms is alkyl­ated (for example, refcode BAHFIZ; Horlein *et al.*, 1981 ▸), the other one is involved in the formation of a doubly hydrogen-bonded dimer. When both nitro­gen atoms are functionalized [refcodes JIMWUY (Hametner *et al.*, 2007 ▸), NAWLIH (Mereiter *et al.*, 2014 ▸), NAWLON *et al.*, 2014 ▸), PILXAK (Hametner *et al.*, 2007 ▸), XAZGAH (Blakemore, *et al.*, 2005 ▸)], no hydrogen-bonds are observed.

**Figure 4:**
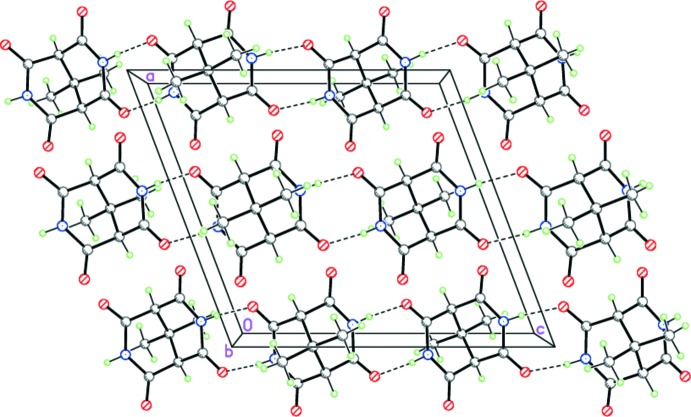
The crystal structure of (I), demonstrating the H-bonded zigzag-like ribbons propagating toward [001]. Dashed lines indicate the inter­molecular N—H⋯O hydrogen bonds.

**Figure 6:**
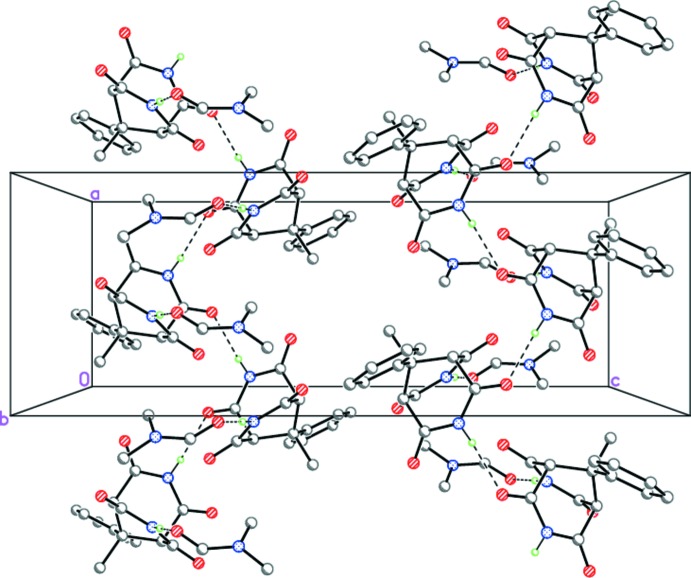
The crystal structure of (III)·DMF, demonstrating the H-bonded zigzag-like chains propagating toward [100]. Dashed lines indicate the inter­molecular N—H⋯O hydrogen bonds.

Despite the geometrical similarity of compounds (I)-(III), they form different supra­molecular structures in the solid state. Thus, in the crystals of (I) and (II), the mol­ecules form the zigzag hydrogen-bonded ribbons by double N—H⋯O hydrogen bonds (Tables 1 ▸ and 2 ▸, Figs. 4 ▸ and 5 ▸). The hydrogen-bonded ribbons in (I) and (II) are distinguished by the binding sites of the 3,7-di­aza­bicyclo­[3.3.1]nonane-2,4,6,8-tetra­one skeleton. According to symmetry, the ribbons in (I) are formed by the two *trans*-arranged O=C—N—H amide fragments, whereas the binding O=C—N—H amide fragments in (II) are *cis* disposed. As one of the two NH groups in (III) is bonded to the dimethyl formamide solvate mol­ecule, the N—H⋯O hydrogen bonds form the zigzag chains rather than ribbons (Table 3 ▸, Fig. 6 ▸).

## Synthesis and crystallization   

**Figure 7:**
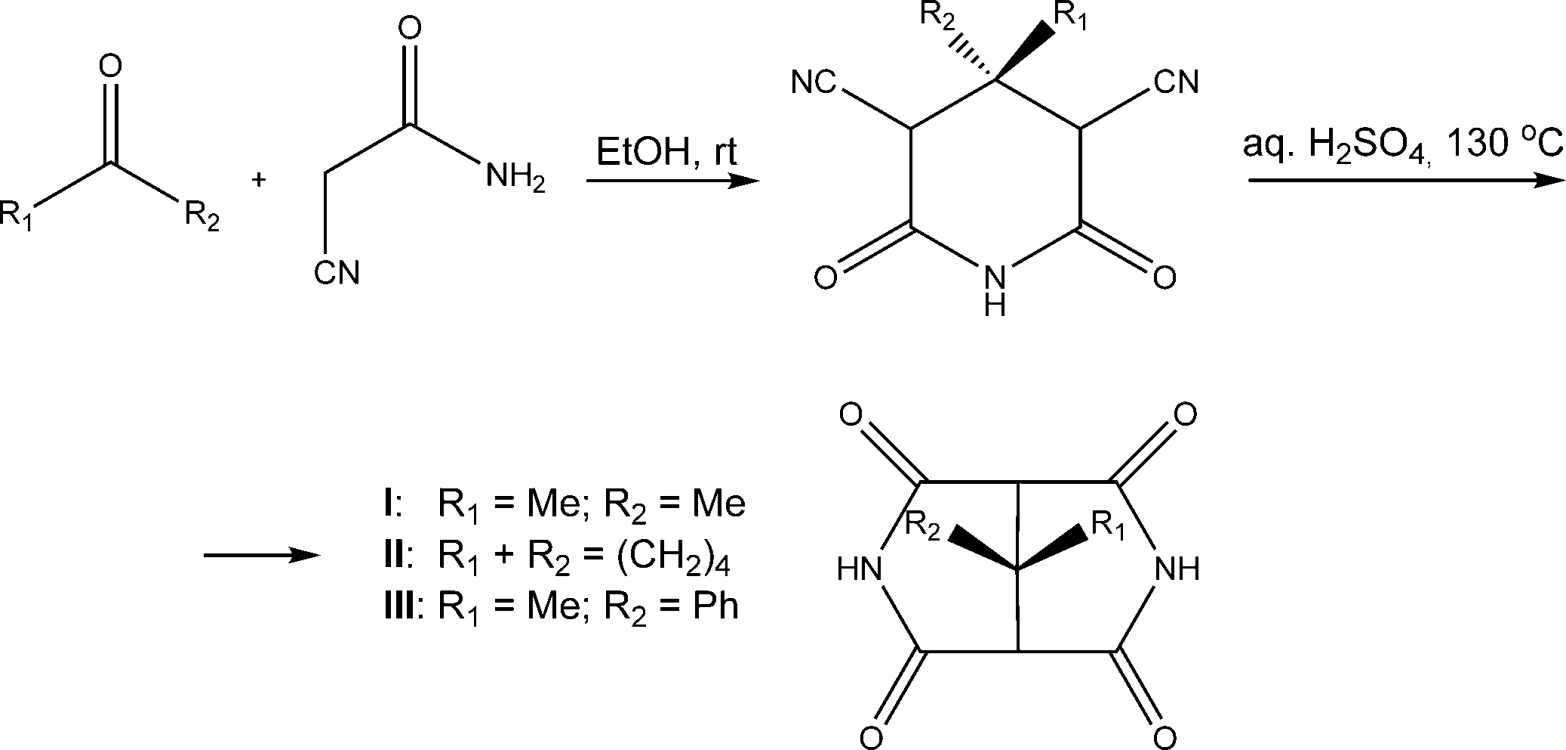
Synthesis of (I)–(III) from 2-cyano­acetamide and ketones.

The title compounds (I)–(III) were synthesized (Fig. 7 ▸) according to the procedure described earlier (Schon *et al.*, 1998 ▸).

Di­nitrile subproducts were obtained by adding 2-cyano­acetamide to the corresponding ketone [(I) – acetone, (II) – aceto­phenone, (III) – cyclo­penta­none] in ethanol at room temperature. Then, the di­nitriles were heated to 393–413 K upon stirring in an acidic medium to complete dissolving. After 10–15 min, the mixture was poured into ice–water. The precipitated tetra­oxo-compounds were filtered off by suction, recrystallized from ethanol solution and finally dried. Single crystals suitable for X-ray diffraction study were obtained by recrystallization of the crude products from DMF solution.

## Refinement   

Crystal data, data collection and structure refinement details are summarized in Table 4 ▸. The hydrogen atoms of the amino groups were localized in the difference-Fourier maps and refined isotropically with fixed displacement parameters [*U*_iso_(H) = 1.2*U*_eq_(N)]. The other hydrogen atoms were placed in calculated positions with C—H = 0.95–1.00 Å and refined in the riding/rotating model with fixed isotropic displacement parameters [*U*_iso_(H) = 1.5*U*_eq_(C) for the CH_3_-groups and 1.2*U*_eq_(C) for the other groups]. The crystal of (I) was a pseudo-merohedral twin. The twin matrix is (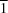 0 0 0 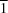 0 0.775 0 1), and BASF = 0.180 (1).

## Supplementary Material

Crystal structure: contains datablock(s) global, I, II, III. DOI: 10.1107/S2056989017009458/ld2140sup1.cif

Structure factors: contains datablock(s) I. DOI: 10.1107/S2056989017009458/ld2140Isup2.hkl

Structure factors: contains datablock(s) II. DOI: 10.1107/S2056989017009458/ld2140IIsup3.hkl

Structure factors: contains datablock(s) III. DOI: 10.1107/S2056989017009458/ld2140IIIsup4.hkl

Supporting information file. DOI: 10.1107/S2056989017009458/ld2140Isup5.cml

Supporting information file. DOI: 10.1107/S2056989017009458/ld2140IIsup6.cml

Supporting information file. DOI: 10.1107/S2056989017009458/ld2140IIIsup7.cml

CCDC references: 1558317, 1558316, 1558315

Additional supporting information:  crystallographic information; 3D view; checkCIF report

## Figures and Tables

**Figure 2:**
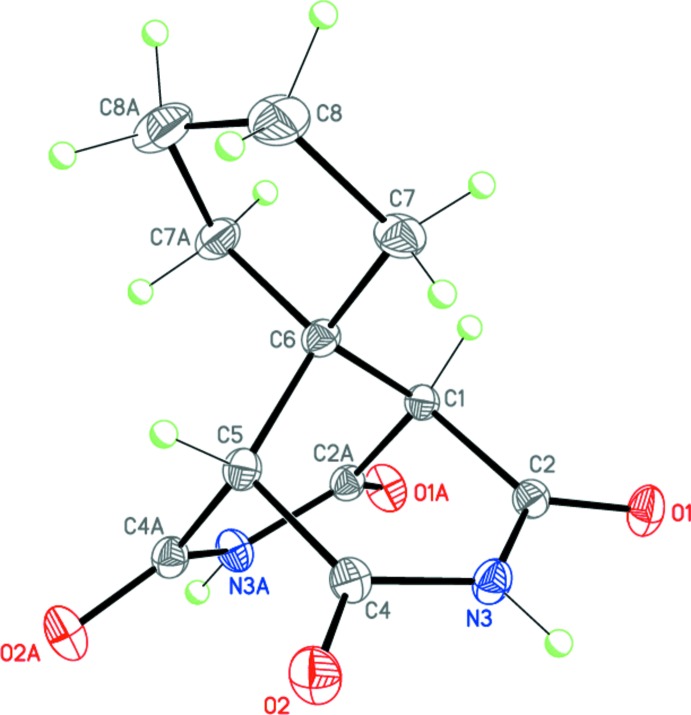
The mol­ecular structure of (II). Displacement ellipsoids are shown at the 50% probability level. H atoms are presented as small spheres of arbitrary radius. [Symmetry code: (A) *x*, 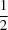 − *y*, *z*.]

**Figure 5:**
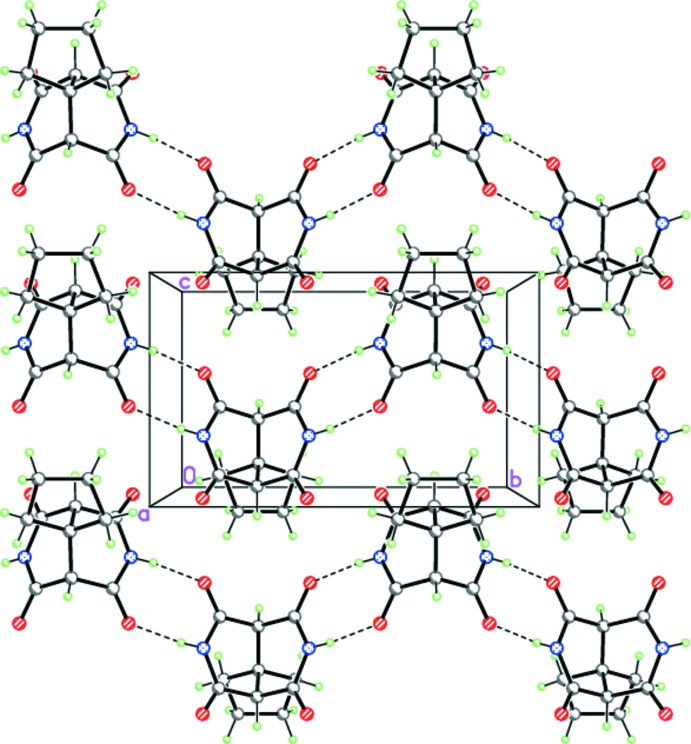
The crystal structure of (II), demonstrating the H-bonded zigzag-like ribbons propagating toward [010]. Dashed lines indicate the inter­molecular N—H⋯O hydrogen bonds.

**Table 1:** Hydrogen-bond geometry (Å, °) for (I)

*D*—H⋯*A*	*D*—H	H⋯*A*	*D*⋯*A*	*D*—H⋯*A*
N3—H3⋯O2^i^	0.90 (3)	2.01 (3)	2.906 (2)	173 (3)

Symmetry code: (i) 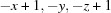.

**Table 2:** Hydrogen-bond geometry (Å, °) for (II)

*D*—H⋯*A*	*D*—H	H⋯*A*	*D*⋯*A*	*D*—H⋯*A*
N3—H3⋯O1^i^	0.855 (14)	2.021 (14)	2.8718 (11)	173.7 (13)

Symmetry code: (i) 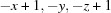.

**Table 3:** Hydrogen-bond geometry (Å, °) for (III)

*D*—H⋯*A*	*D*—H	H⋯*A*	*D*⋯*A*	*D*—H⋯*A*
N3—H3⋯O1^i^	0.861 (19)	2.12 (2)	2.9650 (19)	168.3 (18)
N7—H7⋯O5	0.90 (2)	1.86 (2)	2.7682 (19)	178.6 (18)

Symmetry code: (i) 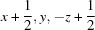.

**Table 4:** Experimental details

	(I)	(II)	(III)
Crystal data			
Chemical formula	C_9_H_10_N_2_O_4_	C_11_H_12_N_2_O_4_	C_14_H_12_N_2_O_4_·C_3_H_7_NO
*M*_r_	210.19	236.23	345.35
Crystal system, space group	Monoclinic, *C*2/*c*	Orthorhombic, *P* *n* *m* *a*	Orthorhombic, *P* *b* *c* *a*
Temperature (K)	100	120	120
*a*, *b*, *c* (Å)	11.4321 (17), 6.6263 (10), 12.4819 (19)	12.8058 (6), 11.4850 (6), 6.9058 (3)	7.7876 (5), 19.4656 (12), 21.7879 (13)
α, β, γ (°)	90, 110.788 (3), 90	90, 90, 90	90, 90, 90
*V* (Å^3^)	884.0 (2)	1015.67 (8)	3302.8 (4)
*Z*	4	4	8
Radiation type	Mo *K*α	Mo *K*α	Mo *K*α
μ (mm^−1^)	0.13	0.12	0.10
Crystal size (mm)	0.30 × 0.20 × 0.15	0.30 × 0.20 × 0.20	0.22 × 0.20 × 0.18

Data collection			
Diffractometer	Bruker *SMART* 1K CCD	Bruker *SMART* 1K CCD	Bruker *SMART* 1K CCD
Absorption correction	Multi-scan (*SADABS*; Sheldrick, 2003 ▸)	Multi-scan (*SADABS*; Sheldrick, 2003 ▸)	Multi-scan (*SADABS*; Sheldrick, 2003 ▸)
*T*_min_, *T*_max_	0.950, 0.970	0.960, 0.970	0.970, 0.975
No. of measured, independent and observed [*I* > 2σ(*I*)] reflections	4993, 1289, 1165	15297, 2181, 1782	41691, 5056, 3210
*R*_int_	0.027	0.031	0.090
(sin θ/λ)_max_ (Å^−1^)	0.703	0.802	0.716

Refinement			
*R*[*F* ^2^ > 2σ(*F* ^2^)], *wR*(*F* ^2^), *S*	0.062, 0.184, 1.06	0.043, 0.119, 1.03	0.051, 0.124, 1.01
No. of reflections	1289	2181	5056
No. of parameters	74	85	235
H-atom treatment	H atoms treated by a mixture of independent and constrained refinement	H atoms treated by a mixture of independent and constrained refinement	H atoms treated by a mixture of independent and constrained refinement
Δρ_max_, Δρ_min_ (e Å^−3^)	0.55, −0.54	0.42, −0.23	0.33, −0.26

Computer programs: *APEX2* (Bruker, 2005 ▸), *SAINT* (Bruker, 2001 ▸), *SHELXT* (Sheldrick, 2015*a* ▸), *SHELXL2014* (Sheldrick, 2015*b* ▸), *SHELXTL* (Sheldrick, 2008 ▸).

## supplementary crystallographic information

### (I) 9,9-Dimethyl-3,7-diazabicyclo[3.3.1]nonane-2,4,6,8-tetraone Crystal data

**Table:**

C_9_H_10_N_2_O_4_	*F*(000) = 440
*M**_r_* = 210.19	*D*_x_ = 1.579 Mg m^−^^3^
Monoclinic, *C*2/*c*	Mo *K*α radiation, λ = 0.71073 Å
*a* = 11.4321 (17) Å	Cell parameters from 3269 reflections
*b* = 6.6263 (10) Å	θ = 3.5–30.0°
*c* = 12.4819 (19) Å	µ = 0.13 mm^−^^1^
β = 110.788 (3)°	*T* = 100 K
*V* = 884.0 (2) Å^3^	Prism, colourless
*Z* = 4	0.30 × 0.20 × 0.15 mm

### (I) 9,9-Dimethyl-3,7-diazabicyclo[3.3.1]nonane-2,4,6,8-tetraone Data collection

**Table:**

Bruker SMART 1K CCD diffractometer	1165 reflections with *I* > 2σ(*I*)
Radiation source: fine-focus sealed tube	*R*_int_ = 0.027
φ and ω scans	θ_max_ = 30.0°, θ_min_ = 1.8°
Absorption correction: multi-scan (SADABS; Sheldrick, 2003)	*h* = −16→16
*T*_min_ = 0.950, *T*_max_ = 0.970	*k* = −9→9
4993 measured reflections	*l* = −17→17
1289 independent reflections	

### (I) 9,9-Dimethyl-3,7-diazabicyclo[3.3.1]nonane-2,4,6,8-tetraone Refinement

**Table:**

Refinement on *F*^2^	Primary atom site location: difference Fourier map
Least-squares matrix: full	Secondary atom site location: difference Fourier map
*R*[*F*^2^ > 2σ(*F*^2^)] = 0.062	Hydrogen site location: mixed
*wR*(*F*^2^) = 0.184	H atoms treated by a mixture of independent and constrained refinement
*S* = 1.06	*w* = 1/[σ^2^(*F*_o_^2^) + (0.0846*P*)^2^ + 3.9571*P*] where *P* = (*F*_o_^2^ + 2*F*_c_^2^)/3
1289 reflections	(Δ/σ)_max_ < 0.001
74 parameters	Δρ_max_ = 0.55 e Å^−^^3^
0 restraints	Δρ_min_ = −0.54 e Å^−^^3^

### (I) 9,9-Dimethyl-3,7-diazabicyclo[3.3.1]nonane-2,4,6,8-tetraone Special details

**Table:**

Geometry. All esds (except the esd in the dihedral angle between two l.s. planes) are estimated using the full covariance matrix. The cell esds are taken into account individually in the estimation of esds in distances, angles and torsion angles; correlations between esds in cell parameters are only used when they are defined by crystal symmetry. An approximate (isotropic) treatment of cell esds is used for estimating esds involving l.s. planes.
Refinement. Refined as a 2-component twin.

### (I) 9,9-Dimethyl-3,7-diazabicyclo[3.3.1]nonane-2,4,6,8-tetraone Fractional atomic coordinates and isotropic or equivalent isotropic displacement parameters (Å^2^)

**Table:**

	*x*	*y*	*z*	*U*_iso_*/*U*_eq_	
C1	0.39535 (15)	0.3325 (3)	0.25497 (14)	0.0103 (4)	
H1	0.3243	0.4158	0.2595	0.012*	
C2	0.43977 (16)	0.1911 (3)	0.35793 (14)	0.0110 (4)	
O2	0.36911 (13)	0.1256 (2)	0.40357 (12)	0.0156 (3)	
N3	0.56404 (14)	0.1339 (2)	0.39595 (13)	0.0119 (4)	
H3	0.591 (3)	0.054 (4)	0.458 (2)	0.014*	
C4	0.65231 (16)	0.1966 (3)	0.35040 (14)	0.0109 (4)	
O4	0.75961 (13)	0.1393 (2)	0.39060 (12)	0.0161 (4)	
C5	0.5000	0.4737 (4)	0.2500	0.0107 (5)	
C6	0.54703 (18)	0.6086 (3)	0.35697 (16)	0.0147 (4)	
H6A	0.6154	0.6941	0.3532	0.022*	
H6B	0.5774	0.5239	0.4257	0.022*	
H6C	0.4783	0.6939	0.3603	0.022*	

### (I) 9,9-Dimethyl-3,7-diazabicyclo[3.3.1]nonane-2,4,6,8-tetraone Atomic displacement parameters (Å^2^)

**Table:**

	*U*^11^	*U*^22^	*U*^33^	*U*^12^	*U*^13^	*U*^23^
C1	0.0104 (7)	0.0099 (8)	0.0096 (7)	−0.0002 (5)	0.0026 (6)	0.0002 (5)
C2	0.0117 (8)	0.0105 (7)	0.0094 (7)	−0.0007 (6)	0.0021 (6)	−0.0012 (6)
O2	0.0148 (6)	0.0192 (7)	0.0135 (6)	−0.0021 (5)	0.0058 (5)	0.0027 (5)
N3	0.0131 (7)	0.0114 (7)	0.0106 (7)	0.0004 (5)	0.0035 (6)	0.0024 (5)
C4	0.0119 (8)	0.0105 (7)	0.0093 (7)	0.0000 (6)	0.0026 (6)	−0.0020 (6)
O4	0.0131 (7)	0.0200 (7)	0.0139 (7)	0.0037 (5)	0.0031 (5)	0.0006 (5)
C5	0.0107 (10)	0.0100 (10)	0.0114 (10)	0.000	0.0039 (8)	0.000
C6	0.0148 (8)	0.0129 (8)	0.0160 (8)	−0.0011 (6)	0.0049 (6)	−0.0039 (6)

### (I) 9,9-Dimethyl-3,7-diazabicyclo[3.3.1]nonane-2,4,6,8-tetraone Geometric parameters (Å, º)

**Table:**

C1—C2	1.525 (2)	N3—H3	0.90 (3)
C1—C4^i^	1.527 (2)	C4—O4	1.210 (2)
C1—C5	1.537 (2)	C5—C6	1.537 (2)
C1—H1	1.0000	C6—H6A	0.9800
C2—O2	1.221 (2)	C6—H6B	0.9800
C2—N3	1.382 (2)	C6—H6C	0.9800
N3—C4	1.386 (2)		
			
C2—C1—C4^i^	105.89 (14)	O4—C4—C1^i^	122.81 (16)
C2—C1—C5	112.14 (13)	N3—C4—C1^i^	116.17 (14)
C4^i^—C1—C5	111.67 (12)	C6^i^—C5—C6	108.8 (2)
C2—C1—H1	109.0	C6—C5—C1^i^	110.59 (9)
C4^i^—C1—H1	109.0	C6—C5—C1	110.87 (10)
C5—C1—H1	109.0	C1^i^—C5—C1	105.1 (2)
O2—C2—N3	120.80 (17)	C5—C6—H6A	109.5
O2—C2—C1	122.27 (16)	C5—C6—H6B	109.5
N3—C2—C1	116.90 (15)	H6A—C6—H6B	109.5
C2—N3—C4	125.92 (15)	C5—C6—H6C	109.5
C2—N3—H3	117.3 (18)	H6A—C6—H6C	109.5
C4—N3—H3	116.7 (18)	H6B—C6—H6C	109.5
O4—C4—N3	120.97 (17)		
			
C4^i^—C1—C2—O2	−86.7 (2)	C2—N3—C4—C1^i^	−3.3 (3)
C5—C1—C2—O2	151.29 (17)	C2—C1—C5—C6^i^	178.06 (14)
C4^i^—C1—C2—N3	91.29 (17)	C4^i^—C1—C5—C6^i^	59.40 (19)
C5—C1—C2—N3	−30.7 (2)	C2—C1—C5—C6	−61.11 (19)
O2—C2—N3—C4	179.35 (16)	C4^i^—C1—C5—C6	−179.77 (14)
C1—C2—N3—C4	1.3 (3)	C2—C1—C5—C1^i^	58.38 (11)
C2—N3—C4—O4	179.26 (16)	C4^i^—C1—C5—C1^i^	−60.28 (11)

Symmetry code: (i) −*x*+1, *y*, −*z*+1/2.

### (I) 9,9-Dimethyl-3,7-diazabicyclo[3.3.1]nonane-2,4,6,8-tetraone Hydrogen-bond geometry (Å, º)

**Table:**

*D*—H···*A*	*D*—H	H···*A*	*D*···*A*	*D*—H···*A*
N3—H3···O2^ii^	0.90 (3)	2.01 (3)	2.906 (2)	173 (3)

Symmetry code: (ii) −*x*+1, −*y*, −*z*+1.

### (II) 3,7-Diazaspiro[bicyclo[3.3.1]nonane-9,1'-cyclopentane]-2,4,6,8-tetraone Crystal data

**Table:**

C_11_H_12_N_2_O_4_	*D*_x_ = 1.545 Mg m^−^^3^
*M**_r_* = 236.23	Mo *K*α radiation, λ = 0.71073 Å
Orthorhombic, *P**n**m**a*	Cell parameters from 4118 reflections
*a* = 12.8058 (6) Å	θ = 3.2–33.9°
*b* = 11.4850 (6) Å	µ = 0.12 mm^−^^1^
*c* = 6.9058 (3) Å	*T* = 120 K
*V* = 1015.67 (8) Å^3^	Prism, colourless
*Z* = 4	0.30 × 0.20 × 0.20 mm
*F*(000) = 496	

### (II) 3,7-Diazaspiro[bicyclo[3.3.1]nonane-9,1'-cyclopentane]-2,4,6,8-tetraone Data collection

**Table:**

Bruker SMART 1K CCD diffractometer	1782 reflections with *I* > 2σ(*I*)
Radiation source: fine-focus sealed tube	*R*_int_ = 0.031
φ and ω scans	θ_max_ = 34.8°, θ_min_ = 3.2°
Absorption correction: multi-scan (SADABS; Sheldrick, 2003)	*h* = −19→19
*T*_min_ = 0.960, *T*_max_ = 0.970	*k* = −17→18
15297 measured reflections	*l* = −10→10
2181 independent reflections	

### (II) 3,7-Diazaspiro[bicyclo[3.3.1]nonane-9,1'-cyclopentane]-2,4,6,8-tetraone Refinement

**Table:**

Refinement on *F*^2^	Primary atom site location: difference Fourier map
Least-squares matrix: full	Secondary atom site location: difference Fourier map
*R*[*F*^2^ > 2σ(*F*^2^)] = 0.043	Hydrogen site location: mixed
*wR*(*F*^2^) = 0.119	H atoms treated by a mixture of independent and constrained refinement
*S* = 1.03	*w* = 1/[σ^2^(*F*_o_^2^) + (0.0631*P*)^2^ + 0.338*P*] where *P* = (*F*_o_^2^ + 2*F*_c_^2^)/3
2181 reflections	(Δ/σ)_max_ < 0.001
85 parameters	Δρ_max_ = 0.42 e Å^−^^3^
0 restraints	Δρ_min_ = −0.23 e Å^−^^3^

### (II) 3,7-Diazaspiro[bicyclo[3.3.1]nonane-9,1'-cyclopentane]-2,4,6,8-tetraone Special details

**Table:**

Geometry. All esds (except the esd in the dihedral angle between two l.s. planes) are estimated using the full covariance matrix. The cell esds are taken into account individually in the estimation of esds in distances, angles and torsion angles; correlations between esds in cell parameters are only used when they are defined by crystal symmetry. An approximate (isotropic) treatment of cell esds is used for estimating esds involving l.s. planes.

### (II) 3,7-Diazaspiro[bicyclo[3.3.1]nonane-9,1'-cyclopentane]-2,4,6,8-tetraone Fractional atomic coordinates and isotropic or equivalent isotropic displacement parameters (Å^2^)

**Table:**

	*x*	*y*	*z*	*U*_iso_*/*U*_eq_	
O1	0.40454 (6)	0.09843 (6)	0.57775 (10)	0.01863 (16)	
O2	0.56727 (6)	0.10479 (6)	−0.00332 (11)	0.02057 (17)	
C1	0.35046 (9)	0.2500	0.35970 (17)	0.0114 (2)	
H1	0.2831	0.2500	0.4330	0.014*	
C2	0.41314 (6)	0.14320 (7)	0.41787 (12)	0.01265 (16)	
N3	0.48223 (6)	0.10060 (7)	0.28341 (11)	0.01450 (16)	
H3	0.5177 (10)	0.0407 (13)	0.3159 (19)	0.017*	
C4	0.49953 (7)	0.14512 (8)	0.09885 (13)	0.01376 (16)	
C5	0.43357 (9)	0.2500	0.04181 (17)	0.0130 (2)	
H5	0.4232	0.2500	−0.1017	0.016*	
C6	0.32636 (9)	0.2500	0.14282 (17)	0.0130 (2)	
C7	0.25898 (8)	0.14463 (9)	0.08181 (14)	0.01871 (19)	
H7A	0.3034	0.0757	0.0571	0.022*	
H7B	0.2078	0.1251	0.1842	0.022*	
C8	0.20315 (9)	0.18259 (12)	−0.10339 (15)	0.0277 (2)	
H8A	0.1307	0.1525	−0.1051	0.033*	
H8B	0.2403	0.1525	−0.2187	0.033*	

### (II) 3,7-Diazaspiro[bicyclo[3.3.1]nonane-9,1'-cyclopentane]-2,4,6,8-tetraone Atomic displacement parameters (Å^2^)

**Table:**

	*U*^11^	*U*^22^	*U*^33^	*U*^12^	*U*^13^	*U*^23^
O1	0.0241 (3)	0.0172 (3)	0.0146 (3)	0.0043 (2)	0.0040 (2)	0.0046 (2)
O2	0.0232 (3)	0.0186 (3)	0.0199 (3)	0.0043 (2)	0.0081 (3)	−0.0004 (3)
C1	0.0116 (4)	0.0102 (4)	0.0124 (4)	0.000	0.0003 (4)	0.000
C2	0.0135 (3)	0.0113 (3)	0.0131 (3)	−0.0001 (3)	0.0003 (3)	0.0000 (3)
N3	0.0172 (3)	0.0123 (3)	0.0140 (3)	0.0038 (2)	0.0023 (2)	0.0015 (2)
C4	0.0160 (4)	0.0120 (3)	0.0133 (3)	−0.0006 (3)	0.0010 (3)	−0.0007 (3)
C5	0.0149 (5)	0.0135 (5)	0.0105 (4)	0.000	−0.0001 (4)	0.000
C6	0.0130 (5)	0.0137 (5)	0.0124 (5)	0.000	−0.0017 (4)	0.000
C7	0.0170 (4)	0.0213 (4)	0.0179 (4)	−0.0047 (3)	−0.0031 (3)	−0.0026 (3)
C8	0.0248 (5)	0.0397 (6)	0.0187 (4)	−0.0066 (4)	−0.0075 (4)	−0.0015 (4)

### (II) 3,7-Diazaspiro[bicyclo[3.3.1]nonane-9,1'-cyclopentane]-2,4,6,8-tetraone Geometric parameters (Å, º)

**Table:**

O1—C2	1.2230 (11)	C5—C6	1.5399 (17)
O2—C4	1.2103 (11)	C5—H5	1.0000
C1—C2	1.5200 (11)	C6—C7	1.5448 (12)
C1—C6	1.5292 (17)	C7—C8	1.5287 (14)
C1—H1	1.0000	C7—H7A	0.9900
C2—N3	1.3727 (11)	C7—H7B	0.9900
N3—C4	1.3910 (11)	C8—C8^i^	1.549 (3)
N3—H3	0.855 (14)	C8—H8A	0.9900
C4—C5	1.5231 (11)	C8—H8B	0.9900
			
C2^i^—C1—C2	107.61 (9)	C1—C6—C5	105.29 (10)
C2—C1—C6	111.43 (6)	C1—C6—C7	112.32 (7)
C2—C1—H1	108.8	C5—C6—C7	111.99 (7)
C6—C1—H1	108.8	C7^i^—C6—C7	103.14 (10)
O1—C2—N3	121.26 (8)	C8—C7—C6	105.43 (9)
O1—C2—C1	122.03 (8)	C8—C7—H7A	110.7
N3—C2—C1	116.69 (8)	C6—C7—H7A	110.7
C2—N3—C4	126.26 (8)	C8—C7—H7B	110.7
C2—N3—H3	116.9 (9)	C6—C7—H7B	110.7
C4—N3—H3	116.8 (9)	H7A—C7—H7B	108.8
O2—C4—N3	120.51 (8)	C7—C8—C8^i^	106.57 (6)
O2—C4—C5	123.33 (9)	C7—C8—H8A	110.4
N3—C4—C5	116.06 (8)	C8^i^—C8—H8A	110.4
C4^i^—C5—C4	104.54 (10)	C7—C8—H8B	110.4
C4—C5—C6	112.17 (7)	C8^i^—C8—H8B	110.4
C4—C5—H5	109.3	H8A—C8—H8B	108.6
C6—C5—H5	109.3		
			
C2^i^—C1—C2—O1	89.14 (11)	C2^i^—C1—C6—C7^i^	−62.03 (11)
C6—C1—C2—O1	−148.42 (9)	C2—C1—C6—C7^i^	177.76 (8)
C2^i^—C1—C2—N3	−89.33 (10)	C2^i^—C1—C6—C7	−177.76 (8)
C6—C1—C2—N3	33.11 (11)	C2—C1—C6—C7	62.03 (11)
O1—C2—N3—C4	−179.14 (8)	C4^i^—C5—C6—C1	−58.65 (7)
C1—C2—N3—C4	−0.66 (13)	C4—C5—C6—C1	58.65 (7)
C2—N3—C4—O2	175.36 (9)	C4^i^—C5—C6—C7^i^	63.69 (12)
C2—N3—C4—C5	−1.04 (13)	C4—C5—C6—C7^i^	−179.00 (8)
O2—C4—C5—C4^i^	−84.18 (12)	C4^i^—C5—C6—C7	179.00 (8)
N3—C4—C5—C4^i^	92.10 (10)	C4—C5—C6—C7	−63.70 (12)
O2—C4—C5—C6	154.04 (9)	C1—C6—C7—C8	155.71 (9)
N3—C4—C5—C6	−29.68 (11)	C5—C6—C7—C8	−86.05 (10)
C2^i^—C1—C6—C5	60.10 (7)	C7^i^—C6—C7—C8	34.55 (12)
C2—C1—C6—C5	−60.11 (7)	C6—C7—C8—C8^i^	−21.58 (8)

Symmetry code: (i) *x*, −*y*+1/2, *z*.

### (II) 3,7-Diazaspiro[bicyclo[3.3.1]nonane-9,1'-cyclopentane]-2,4,6,8-tetraone Hydrogen-bond geometry (Å, º)

**Table:**

*D*—H···*A*	*D*—H	H···*A*	*D*···*A*	*D*—H···*A*
N3—H3···O1^ii^	0.855 (14)	2.021 (14)	2.8718 (11)	173.7 (13)

Symmetry code: (ii) −*x*+1, −*y*, −*z*+1.

### (III) 9-Methyl-9-phenyl-3,7-diazabicyclo[3.3.1]nonane-2,4,6,8-tetraone dimethylformamide monosolvate Crystal data

**Table:**

C_14_H_12_N_2_O_4_·C_3_H_7_NO	*D*_x_ = 1.389 Mg m^−^^3^
*M**_r_* = 345.35	Mo *K*α radiation, λ = 0.71073 Å
Orthorhombic, *P**b**c**a*	Cell parameters from 2856 reflections
*a* = 7.7876 (5) Å	θ = 2.3–26.0°
*b* = 19.4656 (12) Å	µ = 0.10 mm^−^^1^
*c* = 21.7879 (13) Å	*T* = 120 K
*V* = 3302.8 (4) Å^3^	Prism, colourless
*Z* = 8	0.22 × 0.20 × 0.18 mm
*F*(000) = 1456	

### (III) 9-Methyl-9-phenyl-3,7-diazabicyclo[3.3.1]nonane-2,4,6,8-tetraone dimethylformamide monosolvate Data collection

**Table:**

Bruker SMART 1K CCD diffractometer	3210 reflections with *I* > 2σ(*I*)
Radiation source: fine-focus sealed tube	*R*_int_ = 0.090
φ and ω scans	θ_max_ = 30.6°, θ_min_ = 1.9°
Absorption correction: multi-scan (SADABS; Sheldrick, 2003)	*h* = −11→11
*T*_min_ = 0.970, *T*_max_ = 0.975	*k* = −27→27
41691 measured reflections	*l* = −31→30
5056 independent reflections	

### (III) 9-Methyl-9-phenyl-3,7-diazabicyclo[3.3.1]nonane-2,4,6,8-tetraone dimethylformamide monosolvate Refinement

**Table:**

Refinement on *F*^2^	Primary atom site location: difference Fourier map
Least-squares matrix: full	Secondary atom site location: difference Fourier map
*R*[*F*^2^ > 2σ(*F*^2^)] = 0.051	Hydrogen site location: mixed
*wR*(*F*^2^) = 0.124	H atoms treated by a mixture of independent and constrained refinement
*S* = 1.01	*w* = 1/[σ^2^(*F*_o_^2^) + (0.0448*P*)^2^ + 1.1286*P*] where *P* = (*F*_o_^2^ + 2*F*_c_^2^)/3
5056 reflections	(Δ/σ)_max_ < 0.001
235 parameters	Δρ_max_ = 0.33 e Å^−^^3^
0 restraints	Δρ_min_ = −0.26 e Å^−^^3^

### (III) 9-Methyl-9-phenyl-3,7-diazabicyclo[3.3.1]nonane-2,4,6,8-tetraone dimethylformamide monosolvate Special details

**Table:**

Geometry. All esds (except the esd in the dihedral angle between two l.s. planes) are estimated using the full covariance matrix. The cell esds are taken into account individually in the estimation of esds in distances, angles and torsion angles; correlations between esds in cell parameters are only used when they are defined by crystal symmetry. An approximate (isotropic) treatment of cell esds is used for estimating esds involving l.s. planes.

### (III) 9-Methyl-9-phenyl-3,7-diazabicyclo[3.3.1]nonane-2,4,6,8-tetraone dimethylformamide monosolvate Fractional atomic coordinates and isotropic or equivalent isotropic displacement parameters (Å^2^)

**Table:**

	*x*	*y*	*z*	*U*_iso_*/*U*_eq_	
O1	0.91018 (15)	0.32972 (6)	0.24687 (5)	0.0195 (3)	
O2	1.28067 (16)	0.39316 (7)	0.39546 (6)	0.0277 (3)	
O3	1.05101 (18)	0.54938 (6)	0.41859 (6)	0.0284 (3)	
O4	0.71389 (17)	0.48551 (6)	0.25953 (6)	0.0247 (3)	
C1	0.7904 (2)	0.39619 (8)	0.33006 (7)	0.0154 (3)	
H1	0.6777	0.3746	0.3193	0.019*	
C2	0.9331 (2)	0.35860 (8)	0.29593 (7)	0.0156 (3)	
N3	1.09219 (18)	0.36122 (7)	0.32247 (6)	0.0169 (3)	
H3	1.178 (2)	0.3463 (10)	0.3014 (9)	0.020*	
C4	1.1346 (2)	0.39341 (9)	0.37714 (7)	0.0176 (3)	
C5	0.9888 (2)	0.42888 (8)	0.41126 (8)	0.0159 (3)	
H5	1.0146	0.4280	0.4562	0.019*	
C6	0.9822 (2)	0.50346 (9)	0.38999 (8)	0.0197 (4)	
N7	0.89576 (19)	0.51620 (7)	0.33590 (7)	0.0196 (3)	
H7	0.902 (3)	0.5588 (10)	0.3194 (9)	0.024*	
C8	0.7939 (2)	0.46953 (9)	0.30509 (8)	0.0177 (3)	
C9	0.8163 (2)	0.39217 (8)	0.40019 (7)	0.0157 (3)	
C10	0.8217 (2)	0.31744 (8)	0.42225 (7)	0.0157 (3)	
C11	0.7451 (2)	0.26429 (9)	0.38892 (8)	0.0200 (4)	
H11	0.6928	0.2740	0.3505	0.024*	
C12	0.7444 (2)	0.19734 (9)	0.41138 (8)	0.0229 (4)	
H12	0.6912	0.1618	0.3883	0.027*	
C13	0.8208 (2)	0.18232 (9)	0.46704 (8)	0.0226 (4)	
H13	0.8210	0.1365	0.4821	0.027*	
C14	0.8969 (2)	0.23430 (9)	0.50061 (8)	0.0218 (4)	
H14	0.9496	0.2242	0.5389	0.026*	
C15	0.8965 (2)	0.30139 (9)	0.47846 (8)	0.0196 (4)	
H15	0.9482	0.3368	0.5021	0.024*	
C16	0.6721 (2)	0.43004 (9)	0.43480 (8)	0.0205 (4)	
H16A	0.6658	0.4777	0.4205	0.031*	
H16B	0.6965	0.4295	0.4789	0.031*	
H16C	0.5623	0.4071	0.4270	0.031*	
O5	0.91561 (18)	0.64557 (7)	0.28362 (6)	0.0289 (3)	
N1	0.84929 (19)	0.65410 (7)	0.18198 (7)	0.0212 (3)	
C17	0.8439 (2)	0.67440 (9)	0.24001 (8)	0.0232 (4)	
H17	0.7793	0.7146	0.2489	0.028*	
C18	0.9381 (3)	0.59137 (10)	0.16458 (9)	0.0284 (4)	
H18A	1.0181	0.5782	0.1972	0.043*	
H18B	1.0020	0.5991	0.1265	0.043*	
H18C	0.8541	0.5545	0.1583	0.043*	
C19	0.7595 (3)	0.69190 (10)	0.13395 (9)	0.0273 (4)	
H19A	0.6956	0.7301	0.1523	0.041*	
H19B	0.6795	0.6611	0.1128	0.041*	
H19C	0.8431	0.7099	0.1044	0.041*	

### (III) 9-Methyl-9-phenyl-3,7-diazabicyclo[3.3.1]nonane-2,4,6,8-tetraone dimethylformamide monosolvate Atomic displacement parameters (Å^2^)

**Table:**

	*U*^11^	*U*^22^	*U*^33^	*U*^12^	*U*^13^	*U*^23^
O1	0.0215 (6)	0.0208 (6)	0.0164 (6)	0.0008 (5)	−0.0011 (5)	−0.0023 (5)
O2	0.0151 (6)	0.0414 (8)	0.0265 (7)	−0.0016 (6)	−0.0042 (5)	−0.0007 (6)
O3	0.0373 (8)	0.0194 (7)	0.0286 (7)	−0.0081 (6)	−0.0075 (6)	−0.0011 (5)
O4	0.0294 (7)	0.0235 (7)	0.0213 (6)	0.0040 (5)	−0.0068 (5)	0.0021 (5)
C1	0.0142 (8)	0.0162 (8)	0.0160 (8)	−0.0006 (6)	−0.0027 (6)	0.0005 (6)
C2	0.0166 (8)	0.0145 (8)	0.0156 (8)	−0.0004 (6)	0.0002 (6)	0.0030 (6)
N3	0.0127 (7)	0.0211 (7)	0.0168 (7)	0.0026 (6)	0.0007 (5)	0.0004 (6)
C4	0.0176 (8)	0.0189 (8)	0.0162 (8)	−0.0033 (6)	−0.0018 (6)	0.0029 (7)
C5	0.0155 (8)	0.0174 (8)	0.0148 (8)	−0.0029 (6)	−0.0014 (6)	−0.0001 (6)
C6	0.0189 (8)	0.0194 (8)	0.0207 (9)	−0.0007 (7)	−0.0009 (7)	−0.0003 (7)
N7	0.0235 (8)	0.0153 (7)	0.0200 (7)	−0.0010 (6)	−0.0029 (6)	0.0019 (6)
C8	0.0168 (8)	0.0186 (8)	0.0177 (8)	0.0036 (6)	0.0010 (6)	−0.0019 (7)
C9	0.0140 (8)	0.0190 (8)	0.0141 (8)	−0.0015 (6)	−0.0009 (6)	−0.0009 (6)
C10	0.0140 (8)	0.0178 (8)	0.0152 (8)	−0.0023 (6)	0.0037 (6)	0.0002 (6)
C11	0.0221 (9)	0.0217 (9)	0.0162 (8)	−0.0031 (7)	−0.0002 (7)	0.0002 (7)
C12	0.0269 (9)	0.0203 (9)	0.0216 (9)	−0.0052 (7)	0.0030 (7)	−0.0032 (7)
C13	0.0255 (10)	0.0193 (9)	0.0231 (9)	0.0006 (7)	0.0050 (7)	0.0023 (7)
C14	0.0220 (9)	0.0249 (9)	0.0185 (8)	0.0002 (7)	−0.0006 (7)	0.0027 (7)
C15	0.0198 (9)	0.0208 (9)	0.0183 (8)	−0.0028 (7)	−0.0003 (7)	−0.0007 (7)
C16	0.0168 (8)	0.0246 (9)	0.0200 (8)	0.0004 (7)	0.0015 (7)	−0.0014 (7)
O5	0.0420 (8)	0.0229 (7)	0.0218 (7)	−0.0009 (6)	−0.0041 (6)	0.0037 (5)
N1	0.0230 (8)	0.0195 (7)	0.0210 (7)	0.0020 (6)	−0.0005 (6)	0.0007 (6)
C17	0.0286 (10)	0.0169 (8)	0.0240 (9)	−0.0006 (7)	0.0012 (8)	0.0011 (7)
C18	0.0328 (11)	0.0249 (10)	0.0276 (10)	0.0060 (8)	0.0064 (8)	−0.0001 (8)
C19	0.0317 (11)	0.0259 (10)	0.0244 (9)	−0.0014 (8)	−0.0075 (8)	0.0049 (8)

### (III) 9-Methyl-9-phenyl-3,7-diazabicyclo[3.3.1]nonane-2,4,6,8-tetraone dimethylformamide monosolvate Geometric parameters (Å, º)

**Table:**

O1—C2	1.2209 (19)	C11—H11	0.9500
O2—C4	1.205 (2)	C12—C13	1.382 (3)
O3—C6	1.214 (2)	C12—H12	0.9500
O4—C8	1.213 (2)	C13—C14	1.382 (2)
C1—C2	1.524 (2)	C13—H13	0.9500
C1—C8	1.528 (2)	C14—C15	1.392 (2)
C1—C9	1.543 (2)	C14—H14	0.9500
C1—H1	1.0000	C15—H15	0.9500
C2—N3	1.368 (2)	C16—H16A	0.9800
N3—C4	1.386 (2)	C16—H16B	0.9800
N3—H3	0.861 (19)	C16—H16C	0.9800
C4—C5	1.523 (2)	O5—C17	1.237 (2)
C5—C6	1.525 (2)	N1—C17	1.325 (2)
C5—C9	1.541 (2)	N1—C18	1.454 (2)
C5—H5	1.0000	N1—C19	1.458 (2)
C6—N7	1.380 (2)	C17—H17	0.9500
N7—C8	1.380 (2)	C18—H18A	0.9800
N7—H7	0.90 (2)	C18—H18B	0.9800
C9—C10	1.533 (2)	C18—H18C	0.9800
C9—C16	1.540 (2)	C19—H19A	0.9800
C10—C15	1.392 (2)	C19—H19B	0.9800
C10—C11	1.398 (2)	C19—H19C	0.9800
C11—C12	1.392 (2)		
			
C2—C1—C8	105.18 (13)	C12—C11—C10	120.78 (16)
C2—C1—C9	111.30 (13)	C12—C11—H11	119.6
C8—C1—C9	113.43 (13)	C10—C11—H11	119.6
C2—C1—H1	108.9	C13—C12—C11	120.33 (17)
C8—C1—H1	108.9	C13—C12—H12	119.8
C9—C1—H1	108.9	C11—C12—H12	119.8
O1—C2—N3	121.31 (15)	C12—C13—C14	119.61 (17)
O1—C2—C1	122.78 (15)	C12—C13—H13	120.2
N3—C2—C1	115.87 (14)	C14—C13—H13	120.2
C2—N3—C4	126.58 (15)	C13—C14—C15	120.16 (17)
C2—N3—H3	117.7 (13)	C13—C14—H14	119.9
C4—N3—H3	115.2 (13)	C15—C14—H14	119.9
O2—C4—N3	120.51 (16)	C10—C15—C14	121.10 (16)
O2—C4—C5	122.95 (15)	C10—C15—H15	119.5
N3—C4—C5	116.53 (14)	C14—C15—H15	119.5
C4—C5—C6	107.97 (14)	C9—C16—H16A	109.5
C4—C5—C9	111.32 (13)	C9—C16—H16B	109.5
C6—C5—C9	111.39 (14)	H16A—C16—H16B	109.5
C4—C5—H5	108.7	C9—C16—H16C	109.5
C6—C5—H5	108.7	H16A—C16—H16C	109.5
C9—C5—H5	108.7	H16B—C16—H16C	109.5
O3—C6—N7	121.43 (16)	C17—N1—C18	120.96 (15)
O3—C6—C5	122.02 (15)	C17—N1—C19	121.26 (15)
N7—C6—C5	116.55 (15)	C18—N1—C19	117.71 (15)
C6—N7—C8	125.29 (15)	O5—C17—N1	125.66 (17)
C6—N7—H7	118.5 (13)	O5—C17—H17	117.2
C8—N7—H7	116.2 (13)	N1—C17—H17	117.2
O4—C8—N7	121.65 (16)	N1—C18—H18A	109.5
O4—C8—C1	121.45 (15)	N1—C18—H18B	109.5
N7—C8—C1	116.88 (14)	H18A—C18—H18B	109.5
C10—C9—C16	108.70 (13)	N1—C18—H18C	109.5
C10—C9—C5	111.53 (13)	H18A—C18—H18C	109.5
C16—C9—C5	109.69 (13)	H18B—C18—H18C	109.5
C10—C9—C1	111.25 (13)	N1—C19—H19A	109.5
C16—C9—C1	111.43 (13)	N1—C19—H19B	109.5
C5—C9—C1	104.20 (13)	H19A—C19—H19B	109.5
C15—C10—C11	118.01 (15)	N1—C19—H19C	109.5
C15—C10—C9	120.04 (14)	H19A—C19—H19C	109.5
C11—C10—C9	121.86 (14)	H19B—C19—H19C	109.5
			
C8—C1—C2—O1	−88.55 (18)	C4—C5—C9—C16	−179.36 (13)
C9—C1—C2—O1	148.23 (15)	C6—C5—C9—C16	−58.79 (18)
C8—C1—C2—N3	89.02 (16)	C4—C5—C9—C1	−59.96 (16)
C9—C1—C2—N3	−34.20 (19)	C6—C5—C9—C1	60.61 (16)
O1—C2—N3—C4	178.71 (15)	C2—C1—C9—C10	−58.45 (17)
C1—C2—N3—C4	1.1 (2)	C8—C1—C9—C10	−176.82 (13)
C2—N3—C4—O2	−178.77 (16)	C2—C1—C9—C16	−179.93 (13)
C2—N3—C4—C5	0.6 (2)	C8—C1—C9—C16	61.69 (18)
O2—C4—C5—C6	87.7 (2)	C2—C1—C9—C5	61.86 (16)
N3—C4—C5—C6	−91.58 (17)	C8—C1—C9—C5	−56.52 (17)
O2—C4—C5—C9	−149.71 (16)	C16—C9—C10—C15	−77.40 (18)
N3—C4—C5—C9	31.0 (2)	C5—C9—C10—C15	43.7 (2)
C4—C5—C6—O3	−96.53 (19)	C1—C9—C10—C15	159.54 (15)
C9—C5—C6—O3	140.95 (17)	C16—C9—C10—C11	99.18 (17)
C4—C5—C6—N7	82.61 (18)	C5—C9—C10—C11	−139.75 (16)
C9—C5—C6—N7	−39.9 (2)	C1—C9—C10—C11	−23.9 (2)
O3—C6—N7—C8	−170.25 (17)	C15—C10—C11—C12	−0.2 (2)
C5—C6—N7—C8	10.6 (2)	C9—C10—C11—C12	−176.84 (16)
C6—N7—C8—O4	175.77 (16)	C10—C11—C12—C13	−0.4 (3)
C6—N7—C8—C1	−5.8 (2)	C11—C12—C13—C14	0.5 (3)
C2—C1—C8—O4	87.35 (18)	C12—C13—C14—C15	0.0 (3)
C9—C1—C8—O4	−150.80 (15)	C11—C10—C15—C14	0.7 (2)
C2—C1—C8—N7	−91.04 (16)	C9—C10—C15—C14	177.42 (15)
C9—C1—C8—N7	30.8 (2)	C13—C14—C15—C10	−0.6 (3)
C4—C5—C9—C10	60.15 (17)	C18—N1—C17—O5	3.0 (3)
C6—C5—C9—C10	−179.28 (13)	C19—N1—C17—O5	179.89 (18)

### (III) 9-Methyl-9-phenyl-3,7-diazabicyclo[3.3.1]nonane-2,4,6,8-tetraone dimethylformamide monosolvate Hydrogen-bond geometry (Å, º)

**Table:**

*D*—H···*A*	*D*—H	H···*A*	*D*···*A*	*D*—H···*A*
N3—H3···O1^i^	0.861 (19)	2.12 (2)	2.9650 (19)	168.3 (18)
N7—H7···O5	0.90 (2)	1.86 (2)	2.7682 (19)	178.6 (18)

Symmetry code: (i) *x*+1/2, *y*, −*z*+1/2.

## References

[bb1] Blakemore, P. R., Kilner, C., Norcross, N. R. & Astles, P. C. (2005). *Org. Lett.* **7**, 4721–4724. 10.1021/ol0519184 16209519

[bb2] Bruker (2001). *SAINT*. Bruker AXS Inc., Madison, Wisconsin, USA.

[bb3] Bruker (2005). *APEX2*. Bruker AXS Inc., Madison, Wisconsin, USA.

[bb4] Groom, C. R., Bruno, I. J., Lightfoot, M. P. & Ward, S. C. (2016). *Acta Cryst.* B**72**, 171–179. 10.1107/S2052520616003954 PMC4822653 27048719

[bb5] Hametner, C., Dangl, D., Mereiter, K., Marchetti, M. & Fröhlich, J. (2007). *Heterocycles*, **71**, 2331.

[bb6] Horlein, U., Schroder, T. & Born, L. (1981). *Liebigs Ann. Chem.* pp. 1699–1704.

[bb7] Kudryavtsev, K. V., Shulga, D. A., Chupakhin, V. I., Sinauridze, E. I., Ataullakhanov, F. I. & Vatsadze, S. Z. (2014). *Tetrahedron*, **70**, 7854–7864.

[bb8] Medved’ko, A. V., Egorova, B. V., Komarova, A. A., Rakhimov, R. D., Krut’ko, D. P., Kalmykov, S. N. & Vatsadze, S. Z. (2016). *ACS Omega*, **1**, 854-867. 10.1021/acsomega.6b00237 PMC6640746 31457168

[bb17] Mereiter, K., Dangl, D. & Frohlich, J. (2014). Private communication (refcodes NAWLIH, NAWLON). CCDC, Cambridge, England.

[bb9] Norcross, N. R., Melbardis, J. P., Solera, M. F., Sephton, M. A., Kilner, C., Zakharov, L. N., Astles, P. C., Warriner, S. L. & Blakemore, P. R. (2008). *J. Org. Chem.* **73**, 7939–7951. 10.1021/jo8013512 18798673

[bb10] Schön, U., Antel, J., Brückner, R., Messinger, J., Franke, R. & Gruska, A. (1998). *J. Med. Chem.* **41**, 318–331. 10.1021/jm970120q 9464363

[bb11] Sheldrick, G. M. (2003). *SADABS*. Bruker AXS Inc., Madison, Wisconsin, USA.

[bb18] Sheldrick, G. M. (2008). *Acta Cryst.* A**64**, 112–122. 10.1107/S0108767307043930 18156677

[bb12] Sheldrick, G. M. (2015*a*). *Acta Cryst.* A**71**, 3–8.

[bb13] Sheldrick, G. M. (2015*b*). *Acta Cryst.* C**71**, 3–8.

[bb14] Vatsadze, S. Z., Semashko, V. S., Manaenkova, M. A., Krut’ko, D. P., Nuriev, V. N., Rakhimov, R. D., Davlyatshin, D. I., Churakov, A. V., Howard, J. A. K., Maksimov, A. L., Li, W. & Yu, H. (2014). *Russ. Chem. Bull.* **63**, 895–911.

[bb15] Vatsadze, S. Z., Shulga, D. A., Loginova, Y. D., Vatsadze, I. A., Wang, L., Yu, H. & Kudryavtsev, K. V. (2016). *Mendeleev Commun.* **26**, 212–213.

[bb16] Vatsadze, S. Z., Tyurin, V. S., Zyk, N. V., Churakov, A. V., Kuz’mina, L. G., Avtomonov, E. V., Rakhimov, R. D. & Butin, K. P. (2005). *Russ. Chem. Bull.* **54**, 1825–1835.

